# Unicellular Cyanobacteria Are Important Components of Phytoplankton Communities in Australia’s Northern Oceanic Ecoregions

**DOI:** 10.3389/fmicb.2018.03356

**Published:** 2019-01-23

**Authors:** Lisa R. Moore, Taotao Huang, Martin Ostrowski, Sophie Mazard, Sheemal S. Kumar, Hasinika K. A. H. Gamage, Mark V. Brown, Lauren F. Messer, Justin R. Seymour, Ian T. Paulsen

**Affiliations:** ^1^Department of Molecular Sciences, Macquarie University, Sydney, NSW, Australia; ^2^Department of Biological Sciences, University of Southern Maine, Portland, ME, United States; ^3^MQMarine, Macquarie University, Sydney, NSW, Australia; ^4^School of Environmental and Life Sciences, University of Newcastle, Callaghan, NSW, Australia; ^5^Climate Change Cluster, University of Technology Sydney, Sydney, NSW, Australia

**Keywords:** eukaryotic phytoplankton, marine cyanobacteria, Australia, amplicon sequencing, flow cytometry

## Abstract

The tropical marine environments of northern Australia encompasses a diverse range of geomorphological and oceanographic conditions and high levels of productivity and nitrogen fixation. However, efforts to characterize phytoplankton assemblages in these waters have been restricted to studies using microscopic and pigment analyses, leading to the current consensus that this region is dominated by large diatoms, dinoflagellates, and the marine cyanobacterium *Trichodesmium*. During an oceanographic transect from the Arafura Sea through the Torres Strait to the Coral Sea, we characterized prokaryotic and eukaryotic phytoplankton communities in surface waters using a combination of flow cytometry and Illumina based 16S and 18S ribosomal RNA amplicon sequencing. Similar to observations in other marine regions around Australian, phytoplankton assemblages throughout this entire region were rich in unicellular picocyanobacterial primary producers while picoeukaryotic phytoplankton formed a consistent, though smaller proportion of the photosynthetic biomass. Major taxonomic groups displayed distinct biogeographic patterns linked to oceanographic and nutrient conditions. Unicellular picocyanobacteria dominated in both flow cytometric abundance and carbon biomass, with members of the *Synechococcus* genus dominating in the shallower Arafura Sea and Torres Strait where chlorophyll *a* was relatively higher (averaging 0.4 ± 0.2 mg m^-3^), and *Prochlorococcus* dominating in the oligotrophic Coral Sea where chlorophyll *a* averaged 0.13 ± 0.07 mg m^-3^. Consistent with previous microscopic and pigment-based observations, we found from sequence analysis that a variety of diatoms (Bacillariophyceae) exhibited high relative abundance in the Arafura Sea and Torres Strait, while dinoflagellates (Dinophyceae) and prymnesiophytes (Prymnesiophyceae) were more abundant in the Coral Sea. Ordination analysis identified temperature, nutrient concentrations and water depth as key drivers of the region’s assemblage composition. This is the first molecular and flow cytometric survey of the abundance and diversity of both prokaryotic and picoeukaryotic phytoplankton in this region, and points to the need to include the picocyanobacterial populations as an essential oceanic variable for sustained monitoring in order to better understand the health of these important coastal waters as global oceans change.

## Introduction

A combination of prokaryotic and microbial eukaryotic phytoplankton contribute to photosynthetic abundance, biomass and primary production in the oligotrophic and mesotrophic oceans from the temperate latitudes to the tropics. Photosynthetic microbial eukaryotes account for a significant fraction of primary production in temperate mesotrophic waters but can also be important components of subtropical ocean assemblages (Vaulot et al., 2008; Jardillier et al., 2010; Buitenhuis et al., 2012). They are ubiquitous in the surface oceans (Jürgens and Massana, 2008) and their substantial biodiversity contributes to marine ecosystem stability, resilience and function (Loreau et al., 2001; Wardle et al., 2004). Unicellular cyanobacteria are recognized as major photosynthetic prokaryotic players within the microbial food webs and biogeochemical cycles of oligotrophic oceanic regimes (Li, 1994; Partensky et al., 1999; Flombaum et al., 2013). While photosynthetic prokaryotes are important components of coastal and neritic food webs, including regions that support important fisheries, they are not typically included in routine management plans for coastal waters (OzEstuaries, 2003). Understanding the contribution of photosynthetic prokaryotes to the trophic cascade of energy and matter in more productive waters is being recognized as critical to better understanding the health of the oceans (Miloslavich et al., 2018; Ullah et al., 2018).

The diversity and biogeography of microbial phytoplankton across the Northern Australian Shelf provinces (Spalding et al., 2007), from the Arafura Sea through the Torres Strait to the Coral Sea, have not been studied as extensively as other Australian marine provinces (Davies et al., 2016). Yet, these Northern provinces are considered one of the more pristine marine environments on the planet (Halpern et al., 2008) and are culturally and economically significant waters (Condie et al., 2003; Wolanski et al., 2013). The Arafura Sea is a semi-enclosed, shallow (50–80 m), continental shelf basin located between Australia and Indonesian New Guinea, covering about 650,000 km^2^ (Condie and Dunn, 2006) and characterized by high levels of productivity (Condie and Dunn, 2006; Burford et al., 2008). The climate is fully tropical with relatively stable trade winds during part of the year and monsoons typically between November and April (Jongsma, 1974), resulting in strong seasonal upwelling. A combination of deep-water undercurrents, tidal mixing and upwelling result in offshore nutrient enrichment throughout the region (Condie, 2011; Kämpf, 2015, 2016). The Torres Strait is a shallow water body, varying between 7 and 15 m (Harris, 1988), that connects the Arafura Sea to the Coral Sea, which lies between the continental shelf of the Great Barrier Reef and the Gulf of Papua. During the Austral winter (April to November), there are strong prevailing south-east monsoon winds, driving westward flows from the Coral Sea through the Torres Strait to the Arafura Sea (Saint-Cast and Condie, 2006). The Coral Sea is a marginal sea of the South Pacific off the northeast coast of Australia that is relatively deep and stratified, with very low nutrient concentrations in the warm surface waters and low annual primary production (Furnas and Mitchell, 1996; Condie and Dunn, 2006). In this region, the upper 100 m of tropical surface waters splits into two branches on meeting the continental shelf edge, one flowing north along the edge of the Great Barrier Reef, and the other flowing south and contributing to the East Australian Current (Andrews and Clegg, 1989; Lyne et al., 2005).

Reports based on spectrophotometric photosynthetic pigments analysis, fluorescence and electron microscopy suggest that the northern Australian waters are dominated primarily by nanoplankton (2–20 μm), but with distinctly different communities associated with the tropical shelf waters of the Gulf of Carpentaria, dominated by diatoms and the tropical Coral Sea, dominated by dinoflagellates. Some pigment (zeaxanthin and chlorophyll *b*) evidence suggests the presence of small unicellular cyanobacteria in the region (both *Synechococcus* and *Prochlorococcus*; Burford et al., 1995), but although occasional blooms of the filamentous, nitrogen fixing cyanobacteria *Trichodesmium* have been recorded (Hallegraeff and Jeffrey, 1984; Burford et al., 1995; Davies et al., 2016) and linked to high levels of nitrogen fixation (Montoya et al., 2004), there are no records of unicellular cyanobacterial abundance. A recent study analyzing populations using nitrogenase gene (*nifH*) sequence data (Messer et al., 2016) resolved complex spatial and seasonal patterns in cyanobacterial diazotroph dynamics and nitrogen fixation rates in the Arafura/Timor and Coral Seas, suggesting distinctly different physico-chemical and/or biological drivers of cyanobacteria in each water mass, similar, to those previously observed for nanoplankton populations (Hallegraeff and Jeffrey, 1984; McKinnon et al., 2011).

Despite the fact that the molecular level studies of phytoplankton population structure are now routine in many regions of the global oceans (Not et al., 2004; Zhu et al., 2005; Worden and Not, 2008; Zwirglmaier et al., 2008; Mazard et al., 2012; de Vargas et al., 2015; Sohm et al., 2016), there has not been a systematic evaluation of the picocyanobacterial or picoeukaryotic phytoplankton in these important waters. To address this deficiency, in this study we used a high spatial resolution sampling regime and a combination of flow cytometry, high-throughput molecular analysis of both prokaryotic and eukaryotic phytoplankton, and physico-chemical data to further understand the composition and biogeography of surface, pelagic phytoplanktonic assemblages in northern Australian marine ecoregions.

## Materials and Methods

### Microbial Sampling and Oceanographic Data Analysis

Surface seawater samples were collected during a transect through the Arafura Sea, Torres Strait and the Coral Sea on-board RV *Southern Surveyor* in October 2012 (austral spring). A total of 62 water samples were collected from the surface (5 m depth) from both underway (UW) seawater supply (every ∼39 km) and from Niskin bottles attached to a rosette sampling system equipped with a Seabird SBE-911+ Conductivity, Temperature, and Depth (CTD) profiler at selected sites. For each sample, 4 mL of seawater was fixed for 1 h at 41°C with 1% paraformaldehyde (final concentration) before being snap frozen in liquid nitrogen and stored at -80°C (Marie et al., 1997) for later flow cytometry analysis. For microbial community structure analysis, 2 L (UW) or 4–8 L (CTD samples) of seawater were filtered through 0.22 μm pore-size Polyethersulfone filters (Millipore Australia Pty Ltd., North Ryde, NSW, Australia), and filters were flash frozen in liquid nitrogen, then stored at -80°C until DNA extraction.

The concentrations of inorganic nutrients from surface samples collected only from CTD stations were measured on board and reported previously (Messer et al., 2016), but are presented here along the transect presented as geographic distance along the transect. Geographic (Haversine) distances were computed between sampling locations using a least-cost distance strategy as implemented in the “gdistance” R package (van Etten, 2017). For logistical reasons, chlorophyll *a* concentration was only measured at 9 of the 62 sample sites. Thus, in order to get a better understanding of the relationship of phytoplankton populations to the chlorophyll *a* signal across this dynamic region with a consistent set of measurements, we obtained composite chlorophyll *a* for the month of October 2012 from satellite observations (Aqua MODIS, Chlorophyll Concentration OCx, 9 km) downloaded from the ocean color L3 browser^1^. Extracted values were matched to sample stations where possible, and average ± standard deviation was calculated from extracted values at the nearest coordinates between 0.0017 and 0.1 decimal degrees distance (0.017–11 km distant). Only the extracted, composite chlorophyll *a* data is presented.

### Cell Abundance Measurements

Cryopreserved samples were thawed at 34°C and analyzed by flow cytometry within 1 h with minimal exposure to light. We used a Beckman Coulter Cytoflex flow cytometer (Indianapolis, IN, United States) equipped with 50 mW blue laser (488 nm) and followed standard protocols for identifying and quantifying picophytoplankton populations (Marie et al., 2014). Under the settings used, the phytoplankton populations quantified are typically <5 μm representing picocyanobacteria and eukaryotic pico- and ultra-phytoplankton. Although there was no prefiltering done to exclude any size classes for samples analyzed flow-cytometrically, the small volume and the typically lower abundances of larger eukaryotic phytoplankton means that the eukaryotic phytoplankton populations quantified by the flow cytometer are predominantly picoeukaryotic phytoplankton. For ease, we will refer to the flow cytometrically determined eukaryotic phytoplankton as picoeukaryotic phytoplankton throughout the rest of the paper. Biomass of picophytoplankton cells was estimated from flow cytometric cell counts by applying average cellular carbon content (Q_c_) conversion factors for the three populations calculated from literature values found in the Supplementary Table S1.

### DNA Extraction and Sequencing of rRNA Genes

DNA was extracted using the DNeasy PowerWater kit (MoBio Laboratories, Carlsbad, CA, United States) according to the manufacturer’s instructions, with the exception of the addition of a 10 min heating step (60°C) with PW1 prior to bead beating. Eukaryote community composition was assessed by amplicon sequencing of the V9 region of the 18S ribosomal RNA (rRNA) gene for nuclear signatures using barcoded primer set 1380F (5′-TTGTACACACCGCCC-3′) and 1510R (5′-CCTTCYGCAGGTTCACCTAC-3′) (Amaral-Zettler et al., 2009). The barcodes and specific primers for Illumina sequencing using the Nextera Index Kit (Illumina, San Diego, CA, United States) were added to the 5′ ends of the primer pair at the Ramaciotti Centre for Genomics (UNSW Sydney, Australia). PCR amplifications were performed in 50 ml mixtures that contained 0.5 μM of each primer, 2.5 mM MgCl_2_, 1× Buffer (Promega, Madison, WI, United States), 0.2 mM dNTP, 2.5 U Taq DNA polymerase (Qiagen Pty Ltd., Chadstone Centre, VIC, Australia). The reaction conditions consisted of an initial denaturation at 94°C for 3 min before 30 cycles of denaturation at 94°C for 30 s, annealing at 57°C for 60 s, and extension at 72°C for 90 s, then a final extension at 72°C for 10 min. The correct amplicon length of the PCR reaction products was verified via 1.0% agarose gel electrophoresis. The amplicons were pooled and purified using Agencourt AMPure XP beads (Beckman Coulter, Brea, CA, United States) then quantified by Nanodrop ND-2000 (Thermo Fisher Scientific, Waltham, MA, United States) prior to sequencing. Sequencing was done with an Illumina MiSeq platform at the Ramaciotti Centre for Genomics (Sydney, NSW, Australia) using paired-end reads (2 bp × 150 bp for 18S rRNA gene V9 primer set).

Cyanobacterial and chloroplast signatures were determined by sequencing the bacterial V1–V3 region of the 16S rRNA gene using the 27F (5′-AGAGTTTGATCMTGGCTCAG-3′) and 519R (5′-GWATTACCGCGGCKGCTG-3′) barcoded primer set (Lane et al., 1985; Winsley et al., 2012). Barcoding and PCR reactions were done as described for the 18S rRNA gene amplification, except the PCR reaction conditions comprised an initial denaturation step at 95°C for 10 min, and 35 cycles of 95°C for 30 s, 55°C for 10 s, and 72°C for 45 s, with a final extension at 72°C for 5 min. The 16S rRNA PCR products were verified, pooled, and quantified prior to sequencing as described for the 18S rRNA PCR products, and sequenced at the Ramaciotti Centre for Genomics (Sydney, NSW, Australia) with an Illumina MiSeq platform using 2 bp × 250 bp paired-end reads for 16S rRNA gene V1–V3 primer set). For more detailed identification and analysis of the cyanobacterial populations, cyanobacterial 16S–23S rRNA gene internal transcribed spacer (ITS) regions were amplified with barcoded primers 16S 1247F 5′-CGTACTACAATGCTACGG-3′) and 23S 241R (5′-TTCGCTCGCCRCTACT-3′; Rocap et al., 2002). ITS PCR conditions were as described above for the 18S rRNA gene with the exception that annealing was at 51°C. Amplicon sequencing of ITS regions was carried out using 454 GS-FLX titanium at the Ramaciotti Centre for Genomics (Sydney, NSW, Australia).

### Processing Sequencing Reads

Sequence processing and analyses were carried out according to the amplicon sequence analysis workflow of Bissett et al. (2016). Briefly, paired-end reads were joined by FLASH (Magoc and Salzberg, 2011). Merged reads with low Phred quality scores (<20) and lengths shorter than 70 bp for the 18S rRNA gene sequences or less than 200 bp for the 16S rRNA gene sequences were removed. Chimeric sequences were removed *de novo* with USEARCH 64 bit v8.1 (Edgar et al., 2011). The remaining quality filtered, trimmed and joined reads were then clustered into operational taxonomic units (OTUs) using a 97% sequence identify cut-off followed by taxonomic assignments. Taxonomic assignments were performed using mothur classify.seqs (Schloss et al., 2009) with default settings [wang, cut-off = 80 (Kozich et al., 2013)]. Nuclear 18S rRNA gene OTUs were classified against the PR^2^ database (Guillou et al., 2013). The 16S rRNA gene representative OTUs were classified against the Silva123 database (Quast et al., 2013), while 384 16S rRNA gene OTUs identified as chloroplasts (Cyanobacteria;Chloroplasts) were classified against the PhytoRef database (Decelle et al., 2015). The OTUs were filtered to eliminate spurious OTUs by discarding those with <0.005% of the total number of sequences using mothur (Bokulich et al., 2013).

Cyanobacterial ITS sequences were de-multiplexed, denoised, and filtered using mother trim.flows and shhh using default parameters (Schloss et al., 2009). Sequences shorter than 350 nucleotides were discarded and those longer than 450 nucleotides were trimmed and the denoised reads were mapped at 99% identity against 6,212 unique sequences to generate an abundance table. Unique sequences were assigned to clades using the assignTaxonomy and assignSpecies functions in the R package DADA2 (Callahan et al., 2016) using a custom database generated from the cyanobacterial ITS tree from Mazard et al. (2012). Multiple clades were reported where an amplicon sequence had insufficient informative sites to unambiguously assign it to one clade. The Illumina sequence data were deposited in the Sequence Read Archive (SRA-NCBI ^2^) as SRP099130 for the 18S rRNA gene sequences and PRJNA497929 for 16S rRNA gene sequences. The representative 16S–23S rRNA ITS sequences were deposited in figshare^3^.

To get the eukaryote phytoplankton 18S rRNA gene OTU dataset for analysis in this study, each taxon in the 18S rRNA gene OTU dataset was characterized as to its trophic classification [e.g., phototrophic, mixotrophic, and non-phototrophic (i.e., heterotrophic)] according to literature searches (Supplementary Table S1). We then designated the highest taxonomic term that could identify the phototrophic taxa as a term to be used in a global regular expression (grep) command in order to separate the phototrophic and mixotrophic taxa from known non-phototrophs and from those OTUs that could not be determined as phototrophic (i.e., “indeterminant”). For example, since the Stramenopiles class have members that fall within each of the trophic classifications, we designated six terms (*Caecitellus, Leptocylindrus*, MOCH-2, MOCH-5, *Pseudobodo*, and *Talaroneis*) as known to be phototrophic or mixotrophic Stramenopiles, leaving the rest of the Stramenopiles in our dataset as non-phototrophic or indeterminant taxa. Removal of higher plants, non-phototrophs and indeterminant taxa resulted in 1,153 well-defined eukaryote phytoplankton OTU lineages out of 856,467 reads (down from an initial 17,116 OTUs out of 13,503,313 total 18S rRNA gene reads). A list of the 18S rRNA gene taxa, their trophic classifications, literature references, and the terms used to match the regular expressions within the taxonomic lists can be found in Supplementary Table S1.

In addition to separating the chloroplast 16S rRNA gene OTUs into their own dataset for analysis of the eukaryotic phytoplankton that might not have been identified properly in the eukaryotic phytoplankton 18S rRNA gene OTU dataset, we focused our analysis of the 16S rRNA gene OTUs only on the cyanobacteria by removing all non-phototrophic prokaryotic OTUs including the few known non-photosynthetic cyanobacteria, Melainabacteria, ML635J-21 (Soo et al., 2014; Monchampa et al., 2016), and *Candidatus A. thalassa* (Thompson et al., 2012). This left 34 distinct phototrophic cyanobacterial 16S rRNA gene OTU lineages out of 3,598,765 reads (down from 4,209 OTUs out of 8,288,915 total 16S rRNA gene reads). The taxonomic lists of the cyanobacterial 16S rRNA gene OTUs and chloroplast 16S rRNA gene OTUs can be found in the Supplementary Table S1.

### Statistical Data Analysis

A two-tailed, heteroscedastic Student’s *t*-test analysis was used for comparing the oceanographic metadata between the ecoregions. Correlation analysis testing for paired biomass and composite Chl *a* samples, and paired OTU reads and composite Chl *a* samples, was done using cor.test command within the Hmisc package (version 4.1-1^4^). Statistical analysis of community composition and diversity was carried out with R (version 3.4.1) in RStudio (version 1.0.153; RStudio Team, 2016) using the Vegan package (version 2.4-4; Oksanen et al., 2017). Rarefaction curves indicated that the sequencing coverage provided a good representation of the OTU richness for both datasets at both local and regional scales (data not shown). Alpha (Shannon) diversity and richness were calculated on both non-rarefied and rarefied OTU datasets. The trimmed 18S rRNA gene and 16S rRNA gene OTU datasets were aggregated and rarefied to the minimum number of reads per sample site prior to dissimilarities analysis. Dissimilarities between all samples were calculated using a Bray-Curtis dissimilarity matrix. Clustering of communities was carried out using the SIMPROF command in the ClustSig package (Clarke et al., 2008) for each of the 16S rRNA gene and the 18S rRNA gene OTUs. Non-metric Multidimensional Scaling (NMDS) (Minchin, 1987) using the metaMDS function in Vegan was used to visualize the similarity between communities at each site. Environmental factors were fitted to each ordination using the envfit function in Vegan to reveal which local environmental variables correlated with microbial community structure. All metadata was used for envfit analysis except for Province, Lat/Long, Distance along transect, Chl standard deviation, 18S and 16S rRNA gene Shannon diversity index values, and the clusters found for the 18S, 16S, and chloroplast rRNA gene datasets (Supplementary Table S1). Additional multivariate statistical analyses were performed using manyglm model within mvabund package (Wang et al., 2012).

## Results

### Oceanographic Context

**FIGURE 1:**
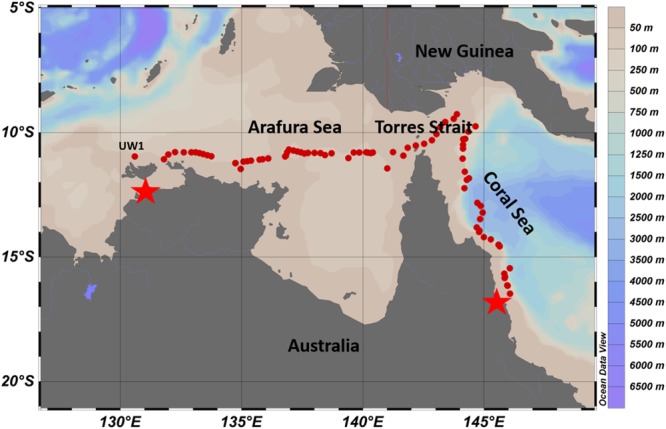
Overview of underway and CTD station sampling sites (red dots) from Darwin (left star) to Cairns (right star) in October 2012. Colors bars on the right indicate values for the bathymetry obtained from Australia Marine National Facility Data portal, http://mnf.csiro.au/Research-outcomes/Data.aspx.

**FIGURE 2:**
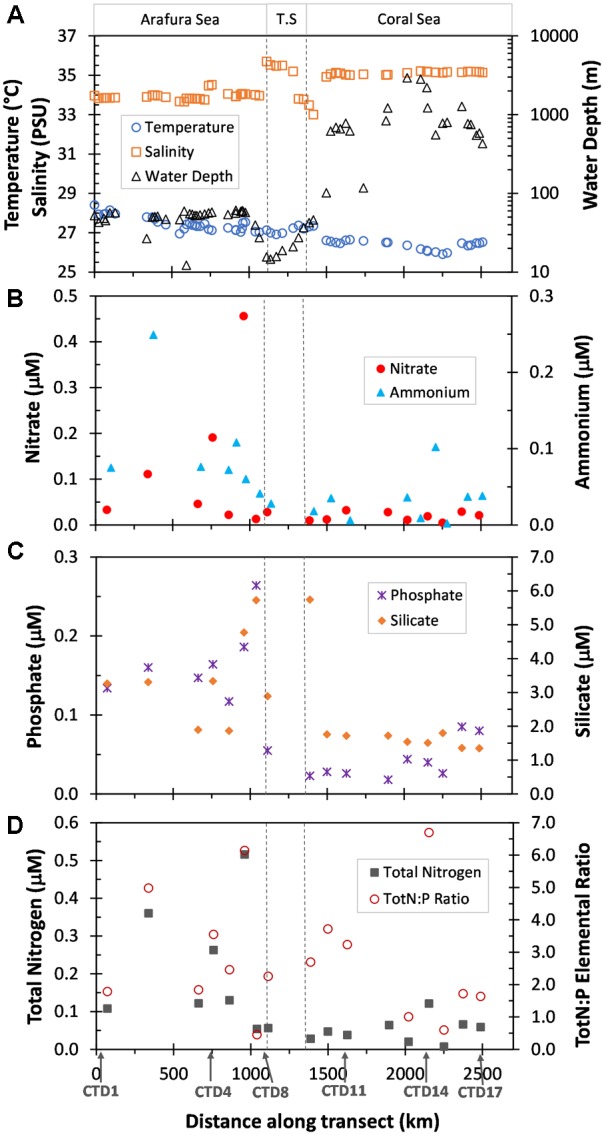
Physical and nutrient characteristics of surface waters sampled along the transect the distance of which was calculated as Haversine distance from first underway sample site. The three ecoregions, Arafura Sea, Torres Strait (T.S.) and Coral Sea, are indicated at the top and delineated by dotted vertical lines. **(A)** Temperature (°C), Salinity (PSU), and depth of the water column (Water Depth, m) obtained from the underway samples. **(B)** Nitrate and Ammonium (μM) measurements from the CTD sample sites. **(C)** Phosphate and Silicate (μM) measurements from the CTD sample sites. **(D)** Calculated Total Nitrogen (TotN, Nitrate + Ammonium, μM) and Total Nitrogen to Phosphorus elemental ratios (TotN:P) for each of the CTD sample sites. Data previously published for each CTD sampling site in Messer et al. (2016).

The research voyage (SS2012_t07) was undertaken in the Austral Spring (October) of 2012 and traversed three distinct ecoregions; the relatively shallow Arafura Sea, the Torres Strait, and the northwestern region of the deep Coral Sea within a range of several kilometers from the outer edge of the Great Barrier Reef (Figure 1; physico-chemical contexts in Supplementary Table S1). The Arafura Sea had a significantly higher sea surface temperature than the much deeper Coral Sea (∼27.5 ± 0.4°C vs. ∼26.4 ± 0.2°C, respectively; *p* < 0.001), while salinity displayed the opposite pattern (∼34.0 ± 0.4 PSU vs. 35.11 ± 0.08 PSU; *p* < 0.001; Figure 2A). The Torres Strait passageway exhibited temperatures closer to that of the Arafura Sea (∼27.2 ± 0.2°C) and displayed large fluctuations in salinity (ranging from a high of 35.6 to a low of 33.3 PSU).

Macronutrients also showed variability in the three ecoregions. Nutrient concentrations (Figures 2B–D) were generally higher [average NO_3_^-^, NH_4_^+^, total Nitrogen, PO_4_^3-^ and silicate were 5.7 (*p* = 0.06), 2.7 (*p* = 0.03), 3.8 (*p* = 0.02), 3.5 (*p* < 0.001), and 2.1 (*p* = 0.03) times higher, respectively] and showed greater fluctuations in the Arafura Sea compared with the Coral Sea. The elemental ratio of inorganic N to PO_4_^3-^ was low and variable across the region, and essentially the same average between the Arafura and Coral Seas (2.9 ± 1.9 vs. 3.3 ± 2.7; Figure 2D).

### Phytoplankton Chlorophyll, Abundance, and Estimated Biomass

**FIGURE 3:**
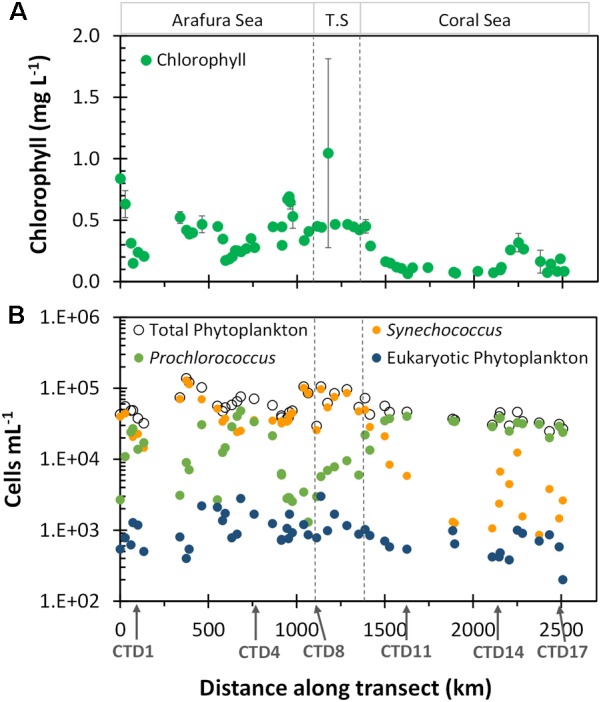
Chlorophyll *a* and flow cytometric cell abundance along the transect. **(A)** Composite monthly average, satellite-derived surface Chlorophyll *a* (μg L^-1^) for October 2012 from NASA’s oceancolor website. Extracted chlorophyll *a* values were matched to sample sites; data with error bars are averaged from values for 2–4 closest available locations relative to the sample sites. **(B)** Phytoplankton cell abundance. Ecoregions as indicated in Figure 2.

The spatial heterogeneity of the surface phytoplankton in the region could be seen by the monthly satellite-derived surface chlorophyll climatology (based on the OC3 algorithm) for the voyage track (Figure 3A and Supplementary Table S1). Overall, the Arafura Sea/Torres Strait region had higher chlorophyll *a* concentrations (0.4 ± 0.2 mg L^-1^) than the oligotrophic Coral Sea (0.13 ± 0.07 mg L^-1^). We also observed hotspots of chlorophyll, that are observed in chlorophyll climatology over more than 5 years (data not shown), and are related to geographic features, such as the high chlorophyll concentration at the beginning of the transect between Melville and Minjilang Islands (sample site UW1, 11.07°S, 131.8°E), and a peak in the western edge of the Torres Strait (sample site UW38, 10.52°S, 142.18°E) which is located close to Thursday Island. South of CTD14 toward the end of the transect in the Coral Sea, stations along the Queensland plateau (Ceccarelli et al., 2013) near the Great Barrier Reef had higher chlorophyll than the northernmost Coral Sea stations.

Flow-cytometrically determined phytoplankton abundances did not differ significantly between the Arafura Sea and the Torres Strait (6.1 × 10^4^ vs. 7.4 × 10^4^ cells mL^-1^, *p* = 0.24) but were slightly higher relative to abundance in the Coral Sea (3.8 × 10^4^ cells mL^-1^, *p* < 0.001; Supplementary Table S1). The picocyanobacteria, including both *Synechococcus* and *Prochlorococcus*, dominated the surface phytoplankton abundance throughout the region (Figure 3B). The picocyanobacterial communities differed distinctly between regions, with *Synechococcus* dominating numbers in the Arafura Sea and Torres Strait regions, while *Prochlorococcus* was more prevalent in the Coral Sea. Picoeukaryotic phytoplankton, identified flow cytometrically by their higher chlorophyll fluorescence and greater side angle light scatter, comprised a measurable but order-of-magnitude lower abundance (Figure 3B), contributing <4% toward the total flow cytometrically observed phytoplankton community cells throughout all regions. The abundance of the picoeukaryotic phytoplankton in the Arafura Sea and Torres Strait was almost double that observed in the Coral Sea (1,170 vs. 625 cells mL^-1^; *p* < 0.01).

**FIGURE 4:**
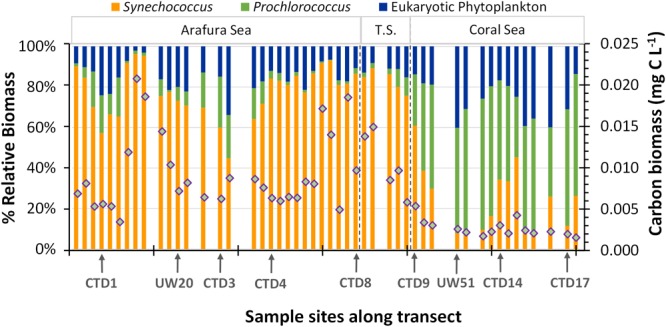
Vertical bars represent the percentage of total carbon biomass estimated from the flow-cytometrically determined eukaryotic, *Synechococcus*, and *Prochlorococcus* phytoplankton groups. The overlaid diamonds represent the total estimated carbon biomass at each sample site.

The difference in cell size of the picocyanobacteria and the picoeukaryotic phytoplankton components within the phytoplankton community means their contribution to energy transfer in the ecosystem differs from what might be predicted from relative abundances. Thus, in order to better understand the relative contributions to primary production in these waters, we calculated the average biomass using cellular carbon content (Q_c_) conversion factors obtained from the literature (for details of calculation see Supplementary Table S1) for each population (*Synechococcus*, 150 ± 91 fg C cell^-1^; *Prochlorococcus*, 39 ± 12 fg C cell^-1^; picoeukaryotic phytoplankton, 2000 ± 1900 fg C cell^-1^) and applied these to the abundances of the three flow-cytometrically measured picophytoplankton populations. We acknowledge that this use of static scalar Q_c_ conversion factors belies the interspecies and environmental variation for biomass within the phytoplankton components (Grob et al., 2007; Casey et al., 2013) but it provides an initial estimate of the relative biomass contribution. Overall, total surface picophytoplankton biomass within the Arafura Sea and Torres Strait regions were the same (0.012 ± 0.005 mg C m^3^ vs. 0.015 ± 0.006 mg C m^3^; *p* = 0.19) and significantly higher (∼threefold; *p* < 0.005) compared to that estimated for the Coral Sea (Figure 4). The highest picophytoplankton biomass could be found in the Arafura Sea (underway sites UW14–UW16) and at the western edge of the Torres Strait, and the lowest across many stations within the Coral Sea. Based on these calculations, the picocyanobacteria populations contributed the most to the picophytoplankton biomass (65%) through the region. *Synechococcus* populations accounted for 60 ± 15% of the biomass and picoeukaryotic phytoplankton accounted for 35 ± 12% in the Arafura Sea and Torres Strait. This situation changed in the Coral Sea where increased *Prochlorococcus* populations made up nearly a third of the biomass on average (30 ± 9%) and markedly reduced *Synechococcus* populations resulted in picoeukaryotic phytoplankton contributing more than half (53 ± 12%).

### Diversity of Phytoplankton Communities

Sequencing of 18S rRNA and 16S rRNA gene amplicons was undertaken to investigate how the respective eukaryotic and prokaryotic phytoplankton assemblages were structured and how this varied at local and regional scales. The 16S rRNA OTU reads is routinely used as a proxy for cyanobacterial population abundances, in large part because there is only 1 copy of the 16S rRNA gene in the majority of *Prochlorococcus* genomes, 2 copies in the majority of marine *Synechococcus* genomes, and 2–5 copies within the other Cyanobacteria Subsections (Stoddard et al., 2015) that are found in much lower abundances, such that error in population abundances determined molecularly is low when compared to their relative abundances as determined flow cytometrically. For eukaryotic phytoplankton, however, the relative abundance of sequences in 18S rRNA gene libraries do not necessarily reflect the absolute abundance of taxa in the original sample due to widely varying rRNA gene copy (Gong et al., 2013). Thus, we have kept the analysis of the 16S rRNA OTU and 18S rRNA OTU datasets discrete. After sequences were clustered into OTUs at 97% similarity and classified for both datasets, higher plants, chloroplasts, non-photosynthetic taxa and those taxa that could not be determined to be photosynthetic (“indeterminant”) were removed (see methods for details), resulting in 1,153 well-defined eukaryote phytoplankton OTU lineages out of 856,467 reads (down from 17,116 OTUs out of 13,503,313 total 18S rRNA gene reads) and 34 distinct phototrophic cyanobacterial 16S rRNA gene OTU lineages out of 3,598,765 reads (down from 4,209 OTUs out of 8,288,915 total 16S rRNA gene reads) (see Supplementary Table S1 for 18S rRNA and 16S rRNA gene phytoplankton and chloroplast OTUs and taxa).

**FIGURE 5:**
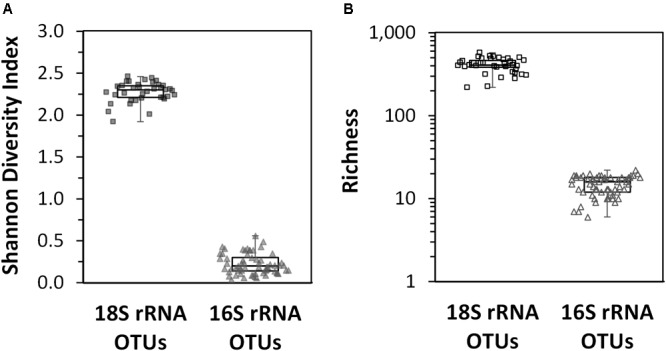
Box plots of diversity indices of the eukaryotic phytoplankton 18S rRNA gene OTUs and cyanobacterial phytoplankton 16S rRNA gene OTU datasets calculated for each sample site across the Arafura Sea, the Torres Strait and the Coral Sea in October 2012. **(A)** Shannon diversity index. **(B)** Richness index.

The Shannon diversity index for the non-rarefied datasets were the same as those calculated for the rarefied datasets (results not shown); thus, only the results for the non-rarefied data is presented here so all sample sites could be used. In surface waters across the entire region, the diversity of eukaryotic phytoplankton OTUs was significantly higher than that observed for the cyanobacterial phytoplankton OTUs (Figure 5A). The highest diversity for the 16S rRNA gene cyanobacterial OTUs was found over the deepest parts of the Arafura Sea north of the Gulf of Carpenteria and in the northwestern part of the Coral Sea. The lowest diversity for 16S rRNA cyanobacterial gene was in surface waters above the deepest parts of the Coral Sea (sample sites CTD12 and CTD13). The richness of the 18S rRNA gene phytoplankton OTUs was 20 times higher than that measured for the 16S rRNA gene cyanobacterial OTUs (Figure 5B). No clear pattern for the phytoplankton 18S rRNA gene diversity or richness could be detected across the region.

### Eukaryotic Phytoplankton Communities

**FIGURE 6:**
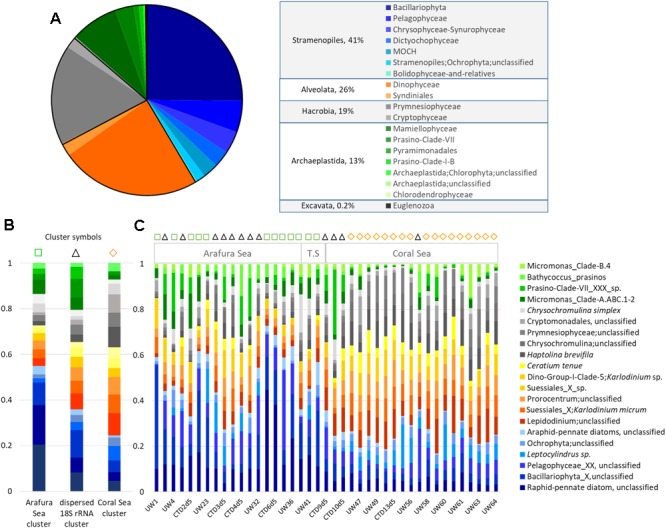
Eukaryotic phytoplankton 18S rRNA gene OTUs community composition. **(A)** Relative abundance of each class, and genera within the most abundant classes. **(B)** Relative abundance of the top 20 18S rRNA gene OTUs at the species level for each of the three Simprof-determined clustered sample sites: Arafura Sea cluster, the “cross-regional” 18S rRNA gene cluster, and the Coral Sea gene cluster. **(C)** Relative abundance for the top 20 18S rRNA species for sample sites across the transect. Simprof clusters for each sample site are indicated by symbols at the top of the barplot: green squares correspond to Arafura Sea/Torres Strait cluster, black triangles to cross-regional cluster, and yellow diamonds to Coral Sea.

An overview of the relative abundance of the eukaryotic phytoplankton 18S rRNA gene OTU dataset revealed 6 phyla (or Super Groups) in these waters, with Stramenopiles contributing 41% of the total 18S rRNA gene OTU abundance, Alveolata 26%, Hacrobia 19%, Archaeplastida 13%, and both Excavata and Rhizaria contributing <1% (Figure 6A). There was one dominant class in each phylum: Bacillariophyta within the Stramenopiles, Dinophyceae within the Alveolata, Prymnesiophyceae within the Hacrobia, and Mamiellophyceae within the Archaeplastida, which together represented close to 75% of the total eukaryotic phytoplankton OTU abundance (25, 24, 17, and 8%, respectively; Figure 6A). Unfortunately, due to the limited resolving power of the V9 hypervariable region of 18S rDNA gene for diatoms (de Vargas et al., 2015), the top 25% of the OTUs were characterized as “unclassified raphid pennate diatoms” and 21% were not even classified at the family-level. The other dominant classes of eukaryotic phytoplankton in these waters could be resolved to the species level. The Dinophyceae class was represented by three genera in almost equal proportions (∼14% each): *Karlodinium micrum, Lepidodinium* spp., and *Prorocentrum* spp., the latter of which were not identified at the species level. *Haptolina brevifila* and *Chrysochromulina* spp. contributed 15 and 14%, respectively, for the Prymnesiophyceae; and *Micromonas* spp. within Clade A represented the majority (31%) of the class Mamiellophyceae.

Similarity profile (SimProf) analysis using Bray-Curtis distance revealed three geographically significant clusters for the eukaryotic phytoplankton OTUs (Figure 6B). Two of the clusters clearly grouped geographically, with the Arafura Sea and Coral Sea; whereas one cluster extended across the entire region (=“cross-region”) (Figure 6C). A condensed look at the distribution of the top 20 most abundant OTUs within each cluster clearly reflects the overall pattern of switching between the dominant taxa in the different ecoregions: the Bacillariophyta class dominating in the Arafura Sea, and the Dinophyceae and Haptophyceae dominating in the Coral Sea. It is interesting to note that the Mamiellophyceae had higher abundances in sample sites corresponding to the “dispersed” cluster. The Arafura Sea and Torres Strait ecoregions show more variability in the proportions of the most abundant eukaryotic phytoplankton, whereas the relative abundances of the major taxa in the Coral Sea sites are more uniformly distributed across the ecoregion (Figure 6C).

Phototrophic eukaryotes were also examined using the chloroplast 16S rRNA gene OTUs. This dataset could not be quantitatively compared to the 18S rRNA gene dataset as the number of taxa found were quite different, due to the lack of Dinophyceae being identified in the chloroplast dataset, the differences in numbers of chloroplast organelles per eukaryotic phytoplankton cells, and differences in 18S rRNA gene copy number (Gong et al., 2013; Mäki et al., 2017). Using two different reference databases to identify and classify chloroplasts (Silva and PhytoRef), we found 18 families and 11 genera that were not identified using the V9 hypervariable region 18S rDNA primers (Supplementary Table S1). Several of these showed differential distributions across the region. For example, the Ochrophyta families, Hemiaulaceae, Naviculaceae, and Vaucheriaceae, were more abundant in the Arafura Sea and Torres Strait. An opposite pattern was observed for the Haptophyte family Hymenomonadaceae and genus *Braarudosphaera*, the latter of which forms a symbiotic relationship with a nitrogen fixing cyanobacterium (*Candidatus A. thalassa* UCYN-A; Thompson et al., 2012; Hagino et al., 2013). Both of these were present in significantly higher abundances in the Coral Sea than in the Arafura Sea and Torres Strait.

### Cyanobacterial Phytoplankton Communities

**FIGURE 7:**
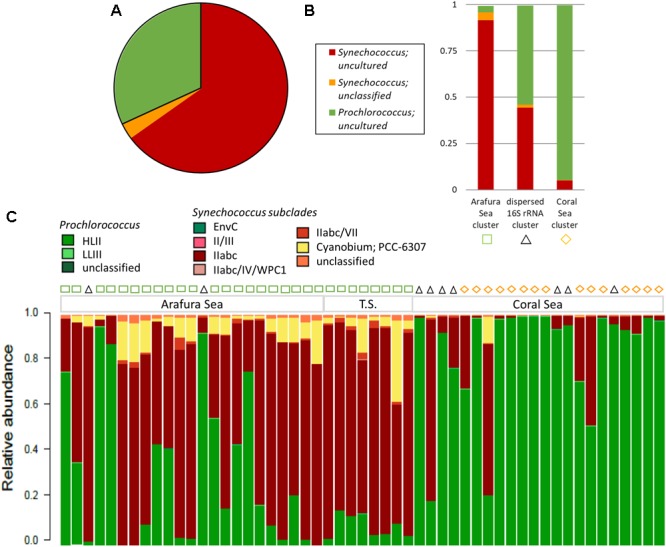
Cyanobacterial phytoplankton community composition. **(A)** Relative abundance of the top 3 cyanobacterial phytoplankton 16S rRNA gene OTUs, which make up 99.5% of the OTU reads. **(B)** Relative abundance for the top 3 cyanobacterial phytoplankton OTUs within the three significant Simprof clusters corresponding to a Arafura Sea cluster, a Coral Sea cluster, and sites that were found throughout the three ecoregions “cross-regional” 16S rRNA gene cluster. **(C)** Relative abundance of the 16S–23S rRNA ITS-based OTU community for sample sites along the transect with the Simprof clusters obtained from the 16S rRNA gene analysis indicated by symbols at the top of the barplot: green squares correspond to Arafura Sea/Torres Strait cluster, black triangles to cross-regional cluster, and yellow diamonds to Coral Sea.

The most abundant cyanobacterial 16S rRNA gene OTUs by far were those belonging to the Cyanobacteria Subsection I genera, *Synechococcus* and *Prochlorococcus*, which made up 99.7% of the total cyanobacterial 16S rRNA gene OTUs in the surface water samples (Figure 7A). Not surprisingly, the majority of the *Synechococcus* and *Prochlorococcus* 16S rRNA gene OTUs were identified as uncultured and unclassified due to low resolution of the 16S rRNA gene V1–V3 region that did not allow accurate identification of the unicellular picocyanobacteria at the strain level. As with the eukaryotic phytoplankton, three significant clusters were found for the cyanobacterial phytoplankton 16S rRNA gene OTUs from SimProf analysis using Bray-Curtis distance (Figure 7B). One cluster clearly grouped within the Arafura Sea and was dominated by uncultured and unclassified *Synechococcus*. A cluster with *Prochlorococcus* dominating corresponded to sites within the Coral Sea. A third cluster was dispersed throughout the ecoregions of these northern waters, though not corresponding to the same sample sites of the “dispersed” 18S rRNA gene cluster, and was characterized by near equal abundances of *Synechococcus* and *Prochlorococcus* 16S rRNA gene OTUs.

Analysis of the relative abundance of the 16S–23S rRNA ITS amplicon sequences, which provide better taxonomic resolution of marine cyanobacteria (Rocap et al., 2002), showed the majority of *Synechococcus* ITS OTUs recruited to subcluster 5.1A, Clade II, specifically WH8109 and CC9605, representing an average of 76.5% of *Synechococcus* ITS OTUs (Figure 7C). Across the Arafura Sea, Subcluster 5.3 ITS OTUs were relatively abundant, representing 23.5% of total *Synechococcus* ITS OTUs, but were virtually absent from the Coral Sea. *Prochlorococcus* genotypes in the region are mostly affiliated with the HLII ecotype lineage as *Prochlorococcus* strain AS9601, representing eMIT9312. Although low in abundance *Prochlorococcus* strain MIT9211, a genotype within the LLII/III ecotype lineage, was detected in several samples sites in the Coral Sea. In addition to the most abundant cyanobacterial genotypes mentioned above, a few members from each of Cyanobacteria Subsection II, III, and IV were also present in these waters (Supplementary Table S1). *Trichodesmium thiebautii* and even a few Oscillatoria, *Leptolyngbya* sp. PCC7376, both from Cyanobacteria Subsection III, Family I, were identified in the 16S rRNA OTU database, primarily in the Arafura Sea and Torres Strait. These filamentous cyanobacteria have been observed previously and are known to be abundant in tropical marine waters of Australia (Marshall, 1933; Revelante and Gilmartin, 1982; Bell, 1992), though not as abundant as the unicellular cyanobacteria.

### Environmental Drivers of Phytoplankton Community Structure

**FIGURE 8:**
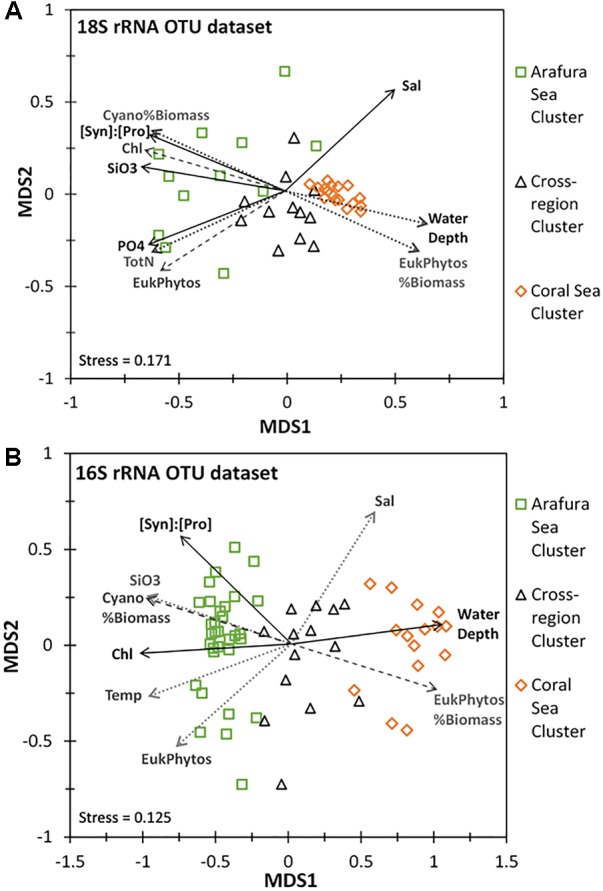
Non-linear multidimensional scaling (NMDS) and environmental factor analyses of the aggregated 18S rRNA and 16S rRNA gene OTU datasets. Only parameters with significant correlation are shown: Salinity (Sal), Chlorophyll *a* (Chl), Temperature (Temp), Water Depth, Total Nitrogen (TotN), Phosphate (PO4), Silicate (SiO3), ratio of *Synechococcus* to *Prochlorococcus* cell abundance ([Syn]:[Pros]), Cyanobacterial % of total estimated biomass (Cyano%Biomass), Eukaryotic phytoplankton’s % of total estimated biomass (EukPhytos%Biomass), Eukaryotic phytoplankton cell abundance (EukPhytos). Line type of environmental factors correspond to *p*-values: solid line, *p* < 0.005; dashed line, *p* < 0.01; dotted line, *p* < 0.05. **(A)** NMDS plot based on Bray-Curtis similarity analysis for the eukaryotic phytoplankton 18S rRNA gene OTU community with the three clusters corresponding to the Arafura Sea cluster, the Coral Sea cluster and the cross-regional cluster. **(B)** NMDS plot based on Bray-Curtis similarity analysis for the cyanobacterial phytoplankton 16S rRNA gene OTU community with three 16S rRNA gene clusters corresponding to the Arafura Sea, the Coral Sea and the cross-regional cluster.

The ordination analysis using non-metric multidimensional scaling calculated with Bray-Curtis distance for the aggregated OTUs at each sample site (16S rRNA gene OTUs were analyzed separately from the 18S rRNA gene OTUs) corresponded well to the similarity profile analysis (Figures 8A,B). Chemical and biological characteristics were strongest determinants for the Arafura Sea cluster. Specifically, the 18S rRNA gene OTUs in Arafura Sea cluster were correlated most strongly with high silicate and high phosphate, low salinity, high levels of chlorophyll, and with high flow cytometric abundance of picoeukaryotic phytoplankton and the ratio of *Synechococcus* abundance relative to *Prochlorococcus* (all *p* < 0.01; Figure 8A). The 16S rRNA gene OTUs in the Arafura Sea cluster were strongly influenced by the ratio of *Synechococcus* abundance relative to *Prochlorococcus*, high chlorophyll and silicate concentrations, but not phosphate (Figure 8B). The Coral Sea cluster for both the 16S and 18S rRNA gene OTUs correlated strongly with depth of the water column (*p* < 0.005). The 18S rRNA gene OTUs within the cross-regional 18S rRNA gene cluster were not clearly associated with any of the chemical, biological, or physical factors within the metadata available for this study. There was more spread in the cyanobacterial 16S rRNA gene OTUs within the cross-regional 16S rRNA gene cluster, with the cyanobacteria in some sample sites associated with higher flow cytometric abundance of picoeukaryotic phytoplankton (Figure 8B).

**FIGURE 9:**
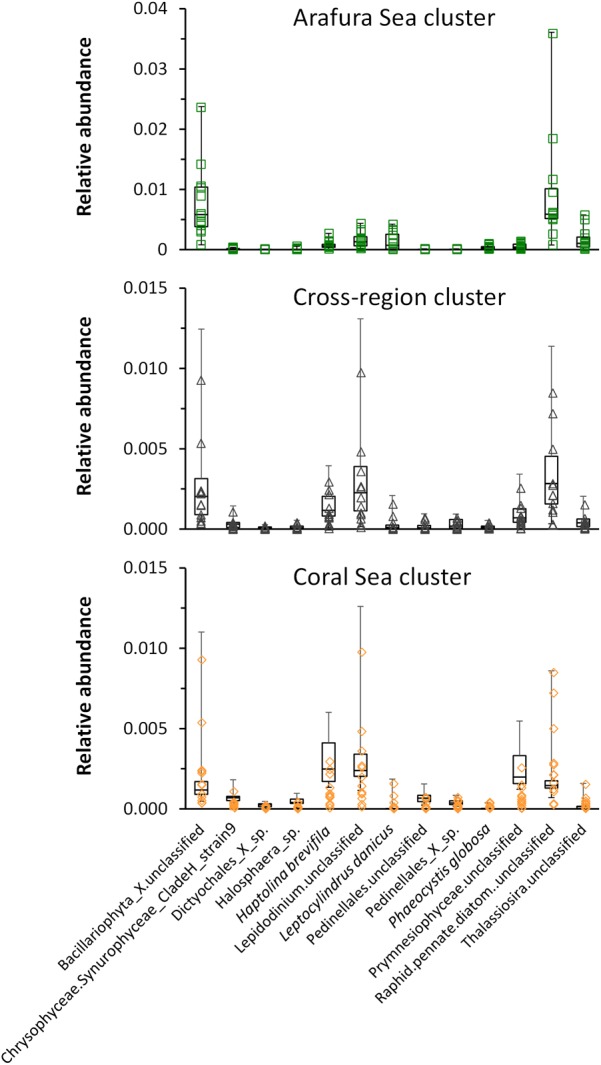
Whisker box plots of relative abundance of the taxa significantly associated (*p* < 0.001) with each of the clusters: Arafura Sea/Torres Strait cluster, Cross-region cluster, and Coral Sea cluster.

In order to test which eukaryotic phytoplankton species might be associated with each sample site, we carried out a multivariate analysis of the aggregated 18S rRNA gene OTU species abundance data [using a negative binomial regression model for each species at each site (manyglm) then testing the output for association with the clusters following methods of Wang et al., 2012]. Thirteen taxa were significantly associated (*p* < 0.001) with each of the clusters but differed in their relative abundance (Figure 9). Raphid pennate diatoms and other unclassified genera of Bacillariophyta were the most significant taxa within the Arafura Sea and the cross-region clusters, with unclassified *Lepidodinium* species showing up as a significant contributor to the cross-region cluster. *Lepidodinium* species extended its significant relative abundance into the Coral Sea cluster where *Haptolina brevifila* and other, unclassified genera within the Prymnesiophyceae, also became significant.

**FIGURE 10:**
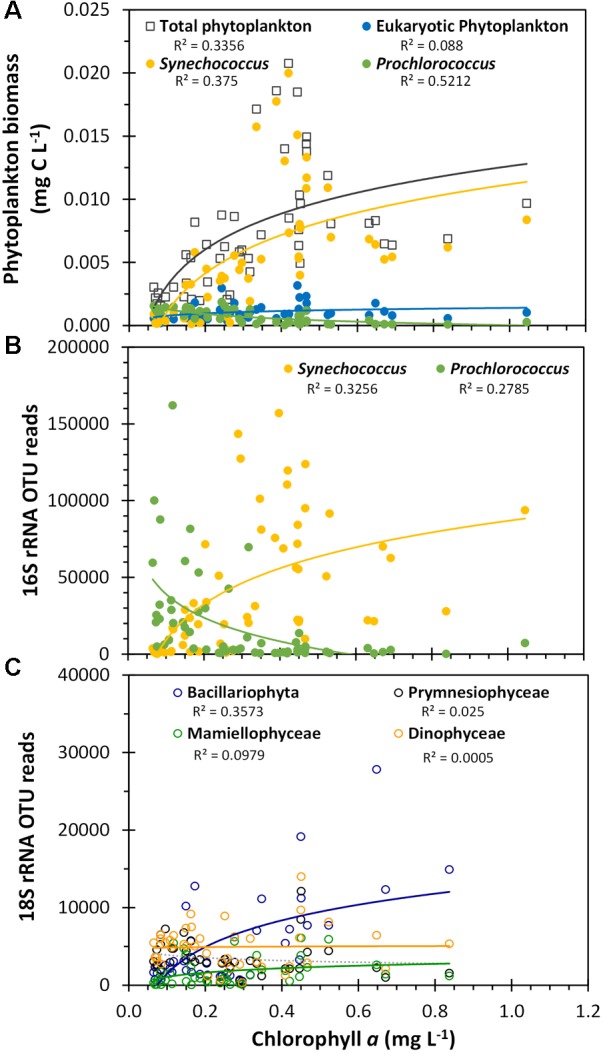
Phytoplankton biomass and OTU reads plotted against satellite-derived, composite average surface chlorophyll *a*. **(A)** Flow cytometric estimated biomass for the three flow cytometrically defined picoplankton populations, as presented in Figure 4. **(B)** Total *Synechococcus* 16S rRNA gene OTU reads and total *Prochlorococcus* 16S rRNA gene OTU reads. **(C)** Class-aggregated 18S rRNA gene OTU reads as shown in Figure 6A. *R*^2^ values are indicated for logarithmic regressions of each phytoplankton group dataset.

The flow cytometric-estimated total picophytoplankton biomass was positively, but non-linearly correlated (Spearman correlation coefficient = 0.69, *p*-value <0.001) with the satellite-derived surface chlorophyll *a* signal across this region, primarily due to the *Synechococcus* population (Figure 10A). We found a significant but negative correlation between *Prochlorococcus* biomass and chlorophyll (Spearman correlation coefficient = -0.75, *p*-value <0.001). These same trends for the two dominant cyanobacterial genera were reflected by the cyanobacterial 16S rRNA gene OTU reads as well (Figure 10B; see Supplementary Table S1 for complete statistical analysis) and were consistent with the ordination analysis where chlorophyll *a* was positively correlated with the *Synechococcus*-dominated cyanobacterial populations in the Arafura Sea cluster and negatively associated with the *Prochlorococcus*-dominated Coral Sea cluster (Figure 8B). When the phytoplankton-specific 18S rRNA gene OTUs were grouped at the class level (Figure 10C), the relative abundance of Bacillariophyta sequences were significantly and positively correlated with satellite-derived surface chlorophyll *a* signal (Pearson correlation coefficient = 0.69, *p*-value <0.001). Again, this is consistent with the ordination analysis showing that satellite-derived surface chlorophyll *a* was positively associated with the phytoplankton 18S rRNA gene Arafura Sea cluster where Bacillariophyta dominate (Figure 8A).

## Discussion

Using a combination of flow cytometry and amplicon-targeted analysis we have provided the most detailed characterization of the abundance and molecular diversity of both eukaryotic and prokaryotic phytoplankton assemblages in surface tropical waters spanning three ecoregions across northern Australia. Cell number estimates showed high abundances and high spatial variability of different picophytoplankton groups particularly through the Arafura Sea and the Torres Strait. Cyanobacteria were the most abundant group in terms of numbers, with *Synechococcus* prominent across the Arafura Sea and the Torres Strait and *Prochlorococcus* more abundant in the Coral Sea. Photosynthetic picoeukaryotes represented a significant but consistently lower proportion of small pigmented cells across the entire region. We acknowledge that the flow cytometric counts of filamentous cyanobacteria and picoeukaryotic phytoplankton in these waters are not fully representative of the total eukaryotic phytoplankton populations for a variety of reason, such as ineffective preservation methods for certain fragile eukaryotic phytoplankton (Vaulot et al., 1989), low abundances and/or capture avoidance for some eukaryotic taxa (Broglio et al., 2001). Despite these caveats, our average flow cytometrically determined abundance of eukaryotic phytoplankton (Figure 3C) matches the maximum abundance of 10^6^ cells L^-1^ (=10^3^ cells mL^-1^) measured previously in these waters (Hallegraeff and Jeffrey, 1984), pointing to the relative accuracy of flow cytometry when the most abundant phytoplankton are small flagellates or nanoplankton.

In addition to similarities in abundance of the picoeukaryotic phytoplankton, our molecular survey of the eukaryotic phytoplankton community revealed distribution patterns with an overall similarity to those previously observed in the region on the basis of morphological and pigment analyses (Hallegraeff and Jeffrey, 1984; Rothlisberg et al., 1994; Burford et al., 1995; Davies et al., 2016). Specifically, we found that Bacillariophyta (diatoms and pelagophytes) continue to be the major eukaryotic phytoplankton group in the Arafura Sea and Torres Strait, and Dinophyta dominate the eukaryotic phytoplankton populations in the Coral Sea (Figure 8A). Ordination analysis of our dataset indicated that higher nutrients are an environmental driver of the eukaryotic phytoplankton in the shallow Arafura Sea, in which the Bacillariophyta dominate, consistent with their global role in biogeochemical cycling in nutrient-rich coastal waters and upwelling areas (Bopp et al., 2005; Armbrust, 2009; Smetacek et al., 2012). However, we observed that some diatoms, such as *Leptocylindrus convexus* and *Leptocylindrus danicus*, which are key components of the phytoplankton in the southeast coastal regions of Australia (Ajani et al., 2016) and in the nearby Darwin National Reference Station (Davies et al., 2016; Brown et al., 2018), were also present in the oligotrophic Coral Sea where they had not been reported previously. This extends the observations of these species across the Arafura Sea and provides evidence for these genetically distinct populations in the adjacent Coral Sea. The Coral Sea cluster of sample sites also showed significant association of *Haptolina brevifila* and other Prymnesiophyceae, consistent with prymnesiophytes being major components of the eukaryotic phytoplankton communities in other open ocean regimes (Moon-van der Staay et al., 2000).

There were some distinct differences in the specific taxa identified using molecular methods compared to previous morphological and pigment analyses. For instance, there were many genera that we did not find in the 18S rRNA gene or chloroplast 16S rRNA gene OTUs, such as the diatom genera *Thalassionema, Thalassiothrix*, and *Bacteriastrum*. These omissions may be due to a variety of factors, such as abundances being too low to be captured in the 2–8 L volumes filtered for DNA, cells being able to avoid the sampling apparatus, and/or the primer set used not being sufficient for resolving all the taxa. The latter of these factors is also a likely reason behind why the highest abundances of OTUs are designated “unclassified” in most of the phyla. The use of additional or different primer sets, such as 18S rRNA gene V4 primers (Bradley et al., 2016), and complementary microscopic and pigment analyses would likely improve the identification of the eukaryotic phytoplankton and provide a more complete assessment of eukaryotic phytoplankton populations.

We found 128 distinct 18S rRNA gene OTUs (Supplementary Table S1) that, to our knowledge, have not been reported previously in these northern Australian waters. For example, we observed the highly abundant and globally distributed genera *Bathycoccus* and *Ostreococcus* within the Mamiellophyceae class of the Archaeplastida phylum, and members of several classes (e.g., Marine Ochrophyta (MOCH), Bolidophyceae and Pinguiophyceae) within the Stramenopiles (Thomsen and Buck, 1998; Honda and Inouye, 2002; Not et al., 2004; Derelle et al., 2006; Vaulot et al., 2008; Worden and Not, 2008; Demir-Hilton et al., 2011; Simmons et al., 2016), that may not have been detected previously by morphological studies due to their small size. Members of the *Azadinium* genus within the class Dinophyceae, observed in these waters for the first time, contain species with the capacity to synthesize azaspiracid toxins associated with shellfish poisoning (Tillmann et al., 2009). This genus was present at multiple sample sites along the transect, but was most abundant in the Coral Sea ecoregion. Although recently reported off New Zealand (Smith et al., 2016), this is the first time this potential toxin-producing genus has been reported in Australian waters and may be a future issue to Australian fisheries.

Chlorophyll *a* is commonly used as a proxy for phytoplankton biomass and primary productivity and is one of the key environmental parameters used for monitoring coastal environments, including around Australia (OzEstuaries, 2003). However, it is essential to consider what contributes to this bulk chlorophyll *a* metric. In this study, the biggest contributor to the satellite-derived surface chlorophyll *a* signal was the *Synechococcus* populations and not the picoeukaryotic phytoplankton. However, we found that the Bacillariophyceae component of the eukaryotic phytoplankton (as determined from 18S rRNA gene OTUs), which dominated in the shallow Arafura Sea and Torres Strait, were correlated with the surface chlorophyll *a* signal. Additionally, *Prochlorococcus* populations were negatively correlated with chlorophyll, particularly within the Coral Sea. This implies that the underlying physical and chemical oceanography of the region plays an important role in structuring some components of the phytoplankton communities. The Arafura Sea is characterized by episodically higher nutrients likely driven by the nutrient rich undercurrent from the West and upwelling as a result of weather-driven turbulence (Condie, 2011; Kämpf, 2015). Additionally, the high PO_4_^3-^ and silicate concentrations and lower salinity near the northeastern edge of the Torres Strait at the northernmost station (station CTD9, -9.45°S, 143.74°E) is likely due to its location near the outflow of the Fly River from Papua New Guinea (Li et al., 2017). This is also consistent with the fact that phosphate and silicate (and total nitrogen, though to a lesser degree) are positively correlated with the eukaryotic phytoplankton in the Arafura Sea cluster. The low inorganic nutrient concentrations measured for the Coral Sea sample sites are consistent with the oligotrophic nature of the deeper Coral Sea (Ceccarelli et al., 2013). Overall, this result shows that there is not always a clear or consistent relationship of surface chlorophyll *a* with specific phytoplankton, biomass and primary productivity, and indicates that the influences of community composition and other environmental factors must be considered.

While eukaryotic phytoplankton are generally thought to be the more important link to secondary production (Vaulot et al., 2008; Buchan et al., 2014) and, hence, transfer to the higher trophic levels, our calculation for the percent contribution to carbon biomass from the flow-cytometrically determined surface picophytoplankton populations indicates that the cyanobacteria contribute as much as 71 ± 12% and picoeukaryotic phytoplankton contribute only 29 ± 12% on average to the carbon biomass in these waters. This calculation is based on a static Q_c_ value applied to the flow cytometric populations because the molecular OTU data cannot be matched with the flow cytometry abundance data; thus, variability of the Q_c_ value for different taxa cannot be determined. However, if we use the average plus 2 standard deviation for the eukaryotic phytoplankton Q_c_ of 5876 fg C cell^-1^ (Supplementary Table S1), the average contribution of the picoeukaryotic phytoplankton would only increase to 52 ± 15%. The point of this estimation is not the specific percent contribution of the eukaryotic or prokaryotic phytoplankton to photosynthetic carbon biomass, but rather that picocyanobacteria are significant contributors to primary production in these waters. This result is consistent with previous findings for nearby regions of the Australian Northwestern Shelf waters, Gulf of Carpenteria and northern Coral Sea (Rothlisberg et al., 1994; Furnas and Mitchell, 1996, 1999). It is still unclear how the cyanobacterial contribution to the carbon biomass and primary production is transferred in this region. Is most of it recycled within the microbial loop (Azam et al., 1983) or transferred to other trophic levels through grazing by small protists and/or larvae, and possibly impacting the fisheries (Apple et al., 2011; Hamilton et al., 2014; Ullah et al., 2018)? This single transect study is the first comprehensive examination of the picophytoplankton in this dynamic region and indicates that the contribution of the smaller members of the phytoplankton assemblage to biomass and primary productivity is necessary for understanding these important northern Australian waters and how the phytoplankton might respond to climate and oceanic changes now and in the future (Miloslavich et al., 2018; Yuan et al., 2018).

## Author Contributions

Study was designed by MO, SM, and IP. MO, LM, MB, and JS collected the samples. LM and TH carried out the flow cytometry measurements. MO, SM, and TH carried out the molecular biology. MO, TH, LM, SK, and HG assisted with bioinformatics and performed statistical analysis. All authors assisted in interpretation of the data and contributed to the discussion of the manuscript. Manuscript was prepared by LM, TH, MO, and IP.

## Conflict of Interest Statement

The authors declare that the research was conducted in the absence of any commercial or financial relationships that could be construed as a potential conflict of interest.

## Footnotes

1 https://oceancolor.gsfc.nasa.gov

2 http://www.ncbi.nlm.nih.gov/sra

3 https://figshare.com/s/ee0bb68c5e20a0a593e0

4 https://CRAN.R-project.org/package=Hmisc

## Supplementary Material

TABLE S1 Metadata, OTU tables for the 18S rRNA gene, 16S rRNA gene, 16S–23S rRNA ITS, and chloroplast 16S rRNA gene, and abundance and biomass data presented in this study for all sample sites across the transect. Additional worksheets provide the list of non-photosynthetic cyanobacterial, non-photosynthetic eukaryotic, and indeterminant eukaryotic taxa removed from the OTU datasets, and the biomass calculations used in estimating the biomass contribution of each phytoplankton group. The first worksheet has a table of contents describing what is in each of the other worksheets.
